# Supplementary material for: Large-angle Lorentz Four-dimensional scanning transmission electron microscopy for simultaneous local magnetization, strain and structure mapping
Source: Nat Commun. 2025 Feb 3;16:1305. doi: 10.1038/s41467-025-56521-6 (PMC11790882; doi:10.1038/s41467-025-56521-6)
Supplement: Supplementary file 1 — Supplementary Information [file 41467_2025_56521_MOESM1_ESM.pdf]

Supplementary materials for "Large-angle Lorentz 4-Dimensional Scanning Transmission Electron Microscopy for Simultaneous Local Magnetization, Strain and Structure Mapping"

Sangjun Kang[1, 2], Maximilian Töllner[1], Di Wang[1, 3], Christian Minnert[4], Karsten Durst[4], Arnaud Caron[5], Rafal E. Dunin-Borkowski[6], Jeffrey McCord[7], Christian Kübel[1, 2, 3*] and Xiaoke Mu[1, 8*]

[1]*Institute of Nanotechnology (INT), Karlsruhe Institute of Technology (KIT), 76344 Eggenstein-Leopoldshafen, Germany*
[2]*In-situ Electron Microscopy, Department of Materials Science, Technical University of Darmstadt (TUDa), 64287 Darmstadt, Germany*
[3]*Karlsruhe Nano Micro Facility (KNMFi), Karlsruhe Institute of Technology (KIT), 76344 Eggenstein-Leopoldshafen, Germany*
[4]*Physical Metallurgy, Department of Materials Science, Technical University of Darmstadt (TUDa), 64287 Darmstadt, Germany*
[5]*Korea University of Technology and Education (Koreatech), 330708 Cheonan, Republic of Korea*
[6]*Ernst Ruska-Centre for Microscopy and Spectroscopy with Electrons, Peter Grünberg Institute, Forschungszentrum Jülich GmbH, 52425 Jülich, Germany.*
[7]*Nanoscale Magnetic Materials – Magnetic Domains, Department of Materials Science, Kiel University, 24143, Kiel, Germany*
[8]*School of Materials and Energy and Electron Microscopy Centre, Lanzhou University, Lanzhou 730000, China*

Supplementary note 1 - details for data processing:

For measuring the ellipticity of the ring, we adopted an algebraic method using singular value decomposition (SVD) as illustrated by Kang et al[1] to fit an ellipse to the diffraction ring. This provides an unbiased fitting method with high precision and orders of magnitude reduced demand for computational power compared to non-linear or iterative fitting methods. We first binarized the diffraction patterns by applying a threshold: Intensity = 1 when $0.95\mathrm{I}_{max} < \mathrm{I}(\mathbf{q_x},\mathbf{q_y}) < \mathrm{I}_{max}$ and Intensity = 0 everywhere else, where I_{max} is the maximum intensity in the diffraction pattern and q_x and q_y are the coordinates of the pixels in reciprocal space. An ellipse in the reciprocal space can be represented by an implicit second-order polynomial equation:

$$\mathbf{F}(\mathrm{C},\mathrm{Q}) = \mathbf{C}\cdot\mathbf{Q} = \mathrm{a}q_x{}^2 + \mathrm{b}q_xq_y + \mathrm{c}q_y{}^2 + \mathrm{d}q_x + \mathrm{e}q_y + \mathrm{f} \quad (1),$$

where $\mathbf{C}$ =[a b c d e f] and $\mathbf{Q} = [q_x{}^2\ q_xq_y\ q_y{}^2\ q_x\ q_y\ 1]^{\mathrm{T}}$. $\mathbf{F}$(C, Q) is the mismatch distance of a

data point (q_x, q_y) to the ellipse $\mathbf{F}(\mathrm{C}, \mathrm{Q}) = 0$. Thus, the best fit of an ellipse to the diffraction ring is equivalent to finding C to minimize the sum of squared mismatch distances $\mathbf{D}(\mathrm{C}) = \min \sum_{i=1}^{N} \mathbf{F}(\mathrm{C}_i, \mathrm{Q}_i)^2$, where N is the total number of selected pixels by the thresholding and i is the pixel sequence number. This least square problem can be solved by singular value decomposition (SVD) considering a rank-deficient generalized eigenvalue system, as $\mathbf{Q}\mathbf{Q}^T\mathbf{C}^T = \lambda\mathbf{P}\mathbf{C}^T$, where $\mathbf{P}$ is a constrain matrix to avoid trivial solutions, e. g. $\mathbf{C} = 0$, as defined by

$$\mathbf{P} = \begin{bmatrix} 0 & 0 & -2 & 0 & 0 & 0 \\ 0 & 1 & 0 & 0 & 0 & 0 \\ -2 & 0 & 0 & 0 & 0 & 0 \\ 0 & 0 & 0 & 0 & 0 & 0 \\ 0 & 0 & 0 & 0 & 0 & 0 \\ 0 & 0 & 0 & 0 & 0 & 0 \end{bmatrix}.$$

Solving the eigenproblem produces six eigenvalues and eigenvectors. The eigenvector associated with the smallest eigenvalue is the $\mathbf{C}$ component minimizing the sum of squared mismatch distances, which is equivalent to the linear least square fitting method. Note that the center position of the diffraction patterns can shift when scanning a large field of view due to the distortion of the beam-focusing lens. Our fitting algorithm simultaneously tracks the centers of the individual diffraction patterns for accurate fitting.

From the fitted ellipse, the relative density was determined as $\Delta\rho = \frac{q_{max}q_{min} - {q_0}^2}{q_{max}q_{min}}$ and the principal strains were determined as $\overrightarrow{\boldsymbol{\varepsilon_1}} = \frac{q_0 - q_{max}}{q_{max}} \begin{pmatrix} \cos(\theta) \\ \sin(\theta) \end{pmatrix}$ and $\overrightarrow{\boldsymbol{\varepsilon_2}} = \frac{q_0 - q_{min}}{q_{min}} \begin{pmatrix} \cos(\theta + 90°) \\ \sin(\theta + 90°) \end{pmatrix}$, where q_0 is the radius of the 1st ring for the unstrained case (averaged from an area far away from the deformed region) and q_{max} and q_{min} are the lengths of the maximum and minimum elliptical axes of the 1st ring. θ is the corresponding azimuthal angle between q_{max} and the x-axis. The deviatoric strain was calculated from both principal strains as $\varepsilon_{dev} = \frac{|\overrightarrow{\varepsilon_1}| - |\overrightarrow{\varepsilon_2}|}{2}$. Although multiple scattering occurs in the thick sample, which reduces the intensity of the diffraction rings and reduces the signal-to-noise level, the intensity of the 1st ring is sufficient for the fitting algorithm. Multiple scattering is an azimuthally isotropic process in amorphous materials within the sampling volume of our experiment. Therefore, it does not create an artificial anisotropy of the diffraction ring.

Supplementary note 2:

The curved feature in the strain and density map shown in Figures 2 a, c, and d arises from thickness during the preparation of the FIB-TEM lamella as seen in Figure S4. To avoid strain relaxation, we conducted a systematic study to see the strain relaxation by reducing the sample thickness by preparing additional FIB-TEM lamella and found the optimized sample thickness for imaging the shear banding feature to be $100<x<200$ nm Figure R3.

The area exhibiting a reduced density in Figure 2 is about 300 nm thick, the situation of which is similar to that shown in the 1st figure in Figure S12a. The apparent density reduction is presumably due to inelastic scattering that happened in the thick sample, which results in a smooth background that is higher in low angles and gradually increases for larger angles. Adding this background shifts the peak position of the 1st diffracted ring slightly resulting in an underestimation of the density. The reduced SNR due to the strong background may reduce the sensitivity for detecting the diffraction ring and reduce the contrast of the shear band, blurring alternations. This may also affect the strain measurement in the thick area. It may be expected that using energy-filtered 4D-STEM can improve the situation. For thinner samples, such as the 200 nm thick sample, the data processing procedure works well and the shear band features are more clearly visible.

Supplementary note 3:

The LA-Ltz-4D-STEM data can provide pair distribution function (PDF) analysis and mapping as an atomic structure descriptor of glasses as shown in Figure S3. In Figure S3a, the temperature-type color corresponds to the amplitude of the PDFs, while the horizontal and vertical axes represent the atomic distance r and the scanning positions on the sample. Shifting features are obvious (in particular at $r \approx 3.4$ and ≈ 4.8 Å) in the shear band region (highlighted in Figure S3 b-d), which divides the PDF line scan into two regions: left and right shear band affected zone (SBAZ). For example, two PDFs from left and right SBAZ are clearly distinguished by their first and second peaks. The difference between PDFs indicates the different atomic arrangements in these zones, which form due to the different strain states. More interestingly, new information in addition to strain effects can be seen in the map of face-share (FS) connected tetrahedral medium-

range order (MRO) (Figure S3e) generated from the STEM-PDF map following the method developed by Mu et al.[2] The shear band in Figure S3e shows darker intensity differing from its two side neighbors and indicates a reduced population of the FS tetrahedra in a shear band.

Supplementary figures:

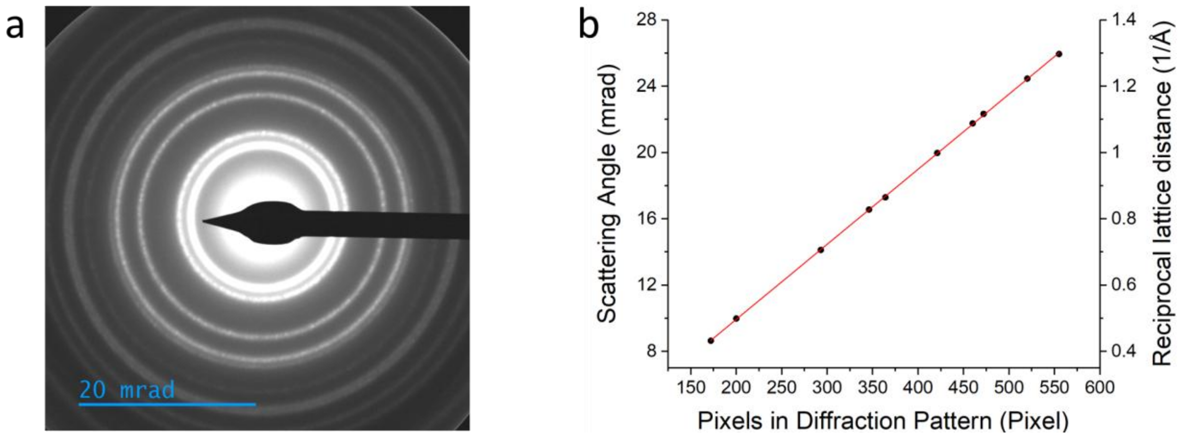

Figure S1: a Diffraction pattern of a standard cross-grating sample in LA-Ltz-4D-STEM mode with reduced C3 lens for creating a large beam size and enough sampling of Au nanoparticles. **b** The diameter of the Au diffraction rings measured on the camera in units of number of pixels versus their theoretical scattering angle for 300 keV electrons. The black dots are experimental data and the red line is a linear fit to the experimental data.

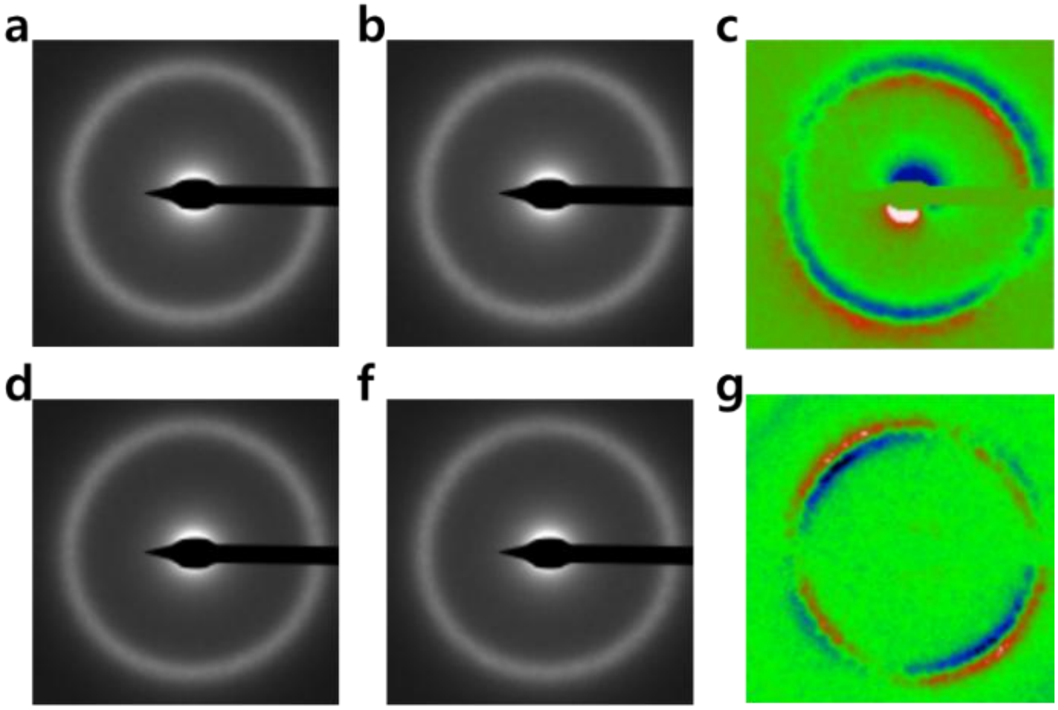

Figure S2: Diffraction patterns acquired by LA-Ltz-4D-STEM from different sample locations. **a** From a domain pointing left-up direction and **b** right-down direction. **c** Subtraction of both **a** and **b** diffraction patterns. **d** Unstrained case, and **f** strained case. **g** Subtraction of both **d** and **f** diffraction patterns

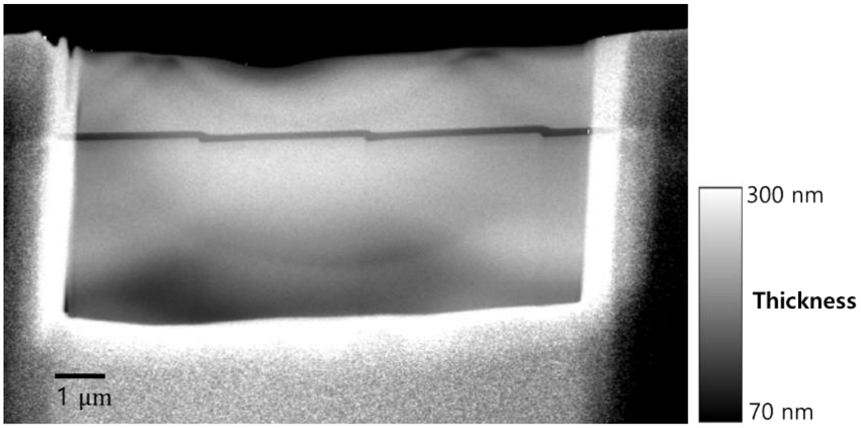

Figure S3: Thickness map of the FIB-prepared lamella from the deformed $Fe_{85.2}Si_{0.5}B_{9.5}P_4Cu_{0.8}$ amorphous metallic alloy obtained using energy-filtered transmission electron microscopy (EFTEM). The mean free path (MFP) of an electron for the material was estimated to be ~75 nm for the thickness estimation.

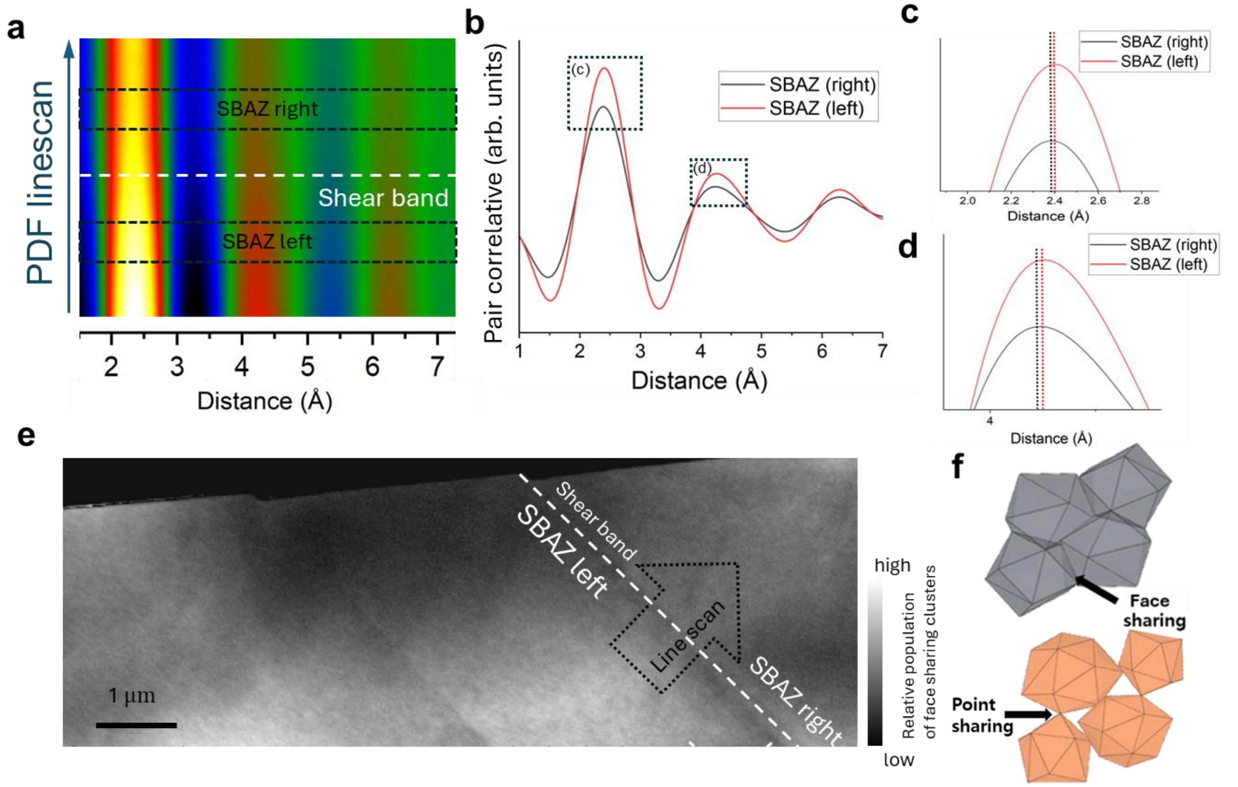

Figure S4. Detailed PDF analysis and map of the edge-shared tetrahedra. **a** PDF line scan following the direction of the arrow in Figure 2e perpendicularly to the shear band. The horizontal axis represents the atomic pair distance r (Å) and the vertical axis corresponds to the scan position on the probed material. The temperature-type color corresponds to the amplitude of the PDFs, that is, the population of pair correlation. **b** PDFs from the left and right shear band affected zone (SBAZ). **c** and **d** are the enlarged parts of the first peak and second peak respectively. **e** Map of face-share (FS) connected tetrahedral medium-range order (MRO) by taking the intensity of PDFs of the STEM-PDF map at r = $8/3R_1, R_1$ is the averaged first peak position of each PDF referring to MD simulation results in Ref.[3]. f Schematic of the basic structural types of polyhedra connected by one shared atom (point sharing) and polyhedra connected by two shared atoms (edge sharing).

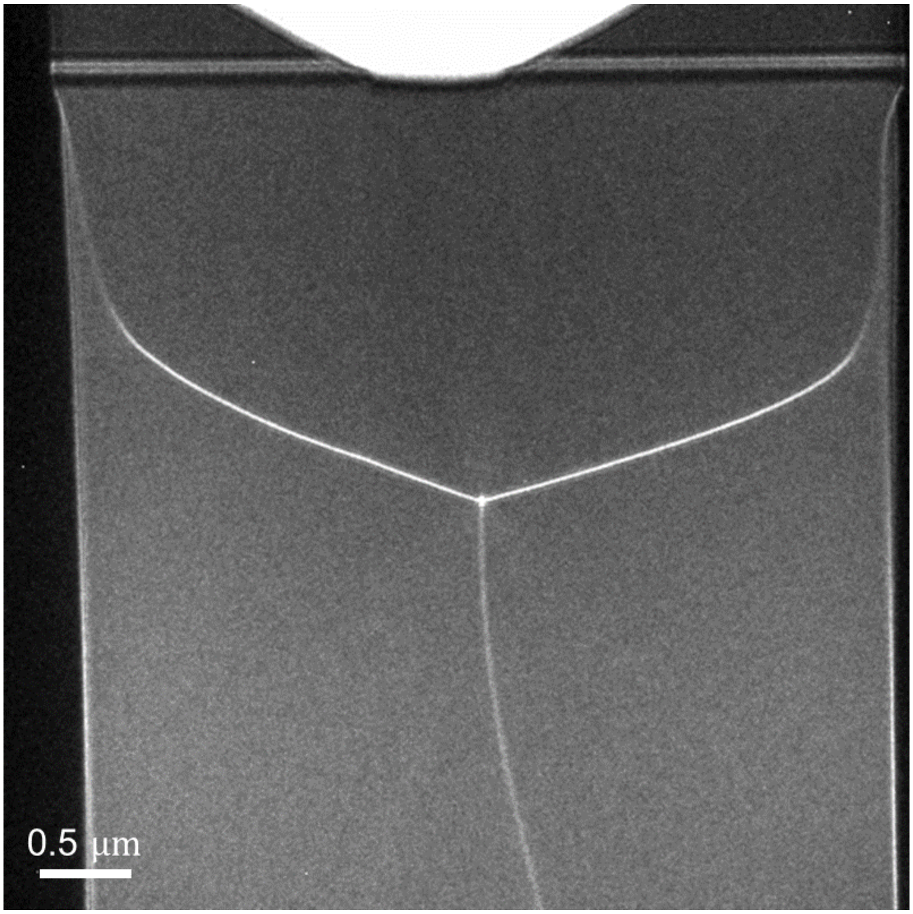

Figure S5: Ltz-TEM images of a TEM lamella of the undeformed $Fe_{85.2}Si_{0.5}B_{9.5}P_4Cu_{0.8}$ amorphous metallic alloy showing the domain structure of the soft ferromagnetic material. Notably, the undeformed sample may also possess some degree of residual stress anisotropy from fast melt-spin quenching, leading to the observed variation of the magnetic domain structure from a regular Landau-like domain structures, reflected in the curvature of the domain walls and their variation from a regular closure domain angle.

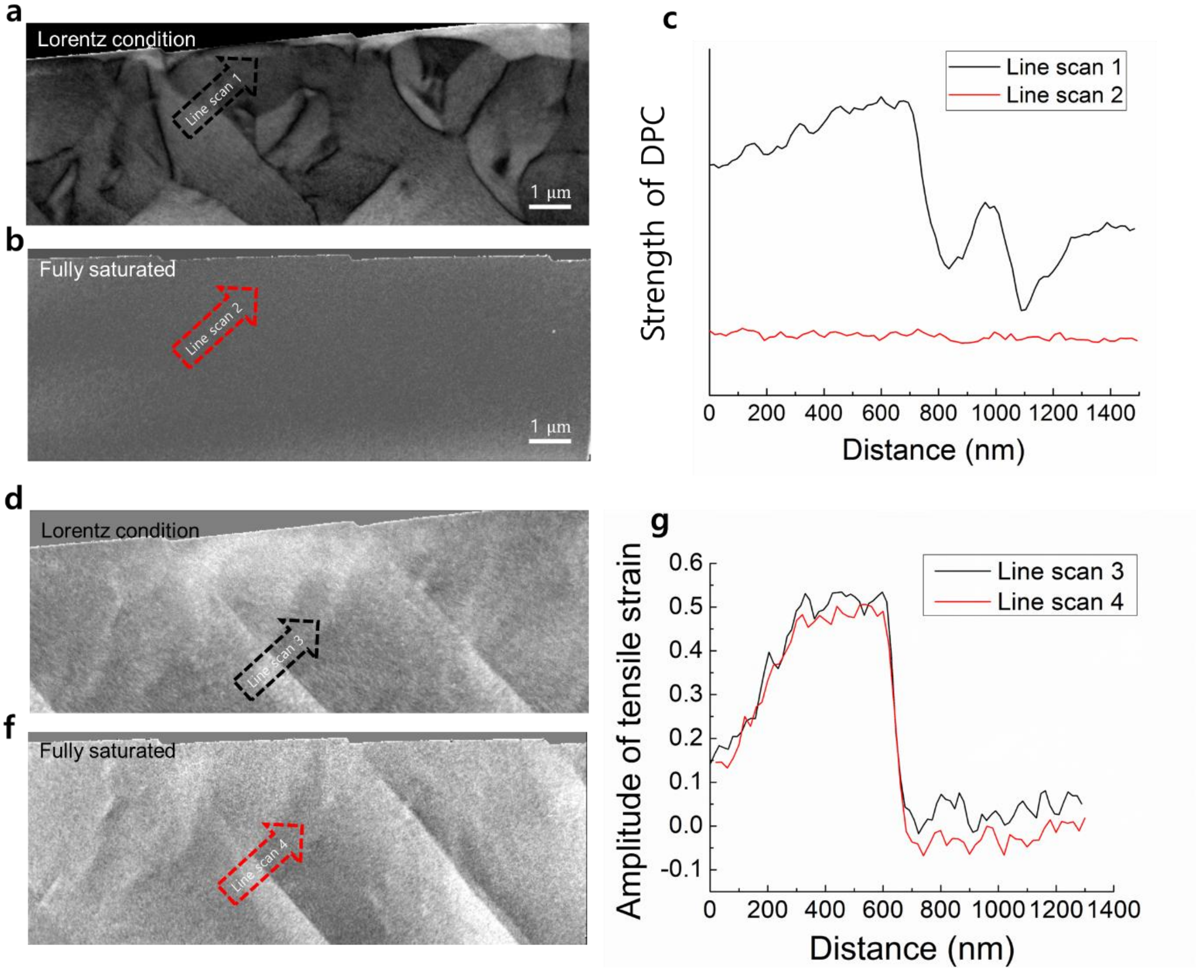

Figure S6: Map of the amplitude of the in-plane magnetization of the deformed amorphous $Fe_{85.2}Si_{0.5}B_{9.5}P_4Cu_{0.8}$ metallic alloy at **a** unmagnetized state (field-free condition), and **b** fully vertically magnetized state (conventional microprobe STEM condition with objective excited about 2 T). **c** Line profiles taken across the shear band. Line scan 1: From the image of **a** along the black dash arrow. Line scan 2: From **b** along the red dash arrow. Map of the amplitude of the tensile strain at **d** unmagnetized state (field-free condition), and **f** fully vertically magnetized state. **g** Line profiles taken across the shear band. Line scan 3: From the image of **d** along the black dash arrow. Line scan 4: From **f** along the red dash arrow.

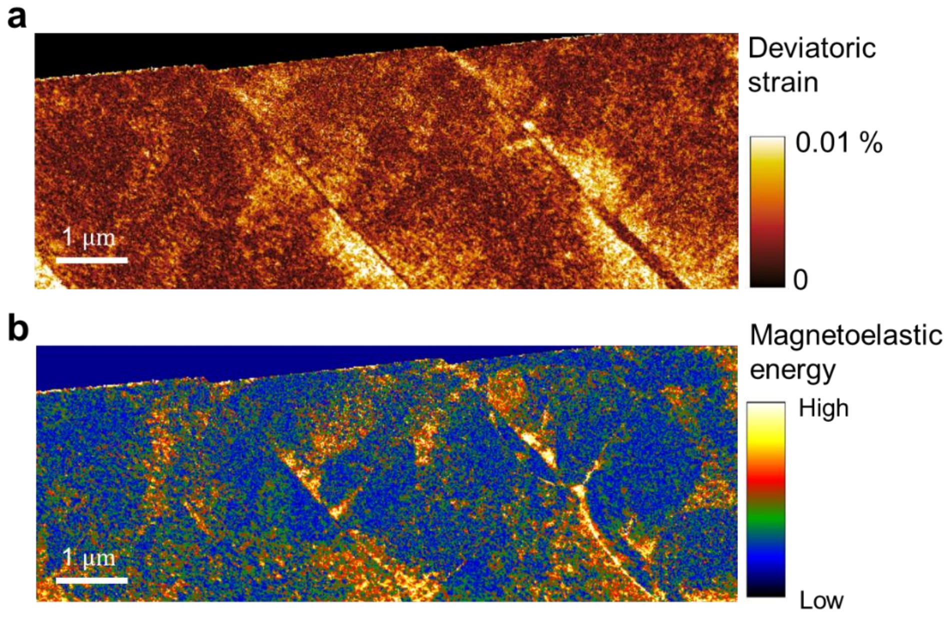

Figure S7: a Map of the deviatoric strain of the plastically deformed amorphous metallic alloy. **b** Map of magnetoelastic energy.

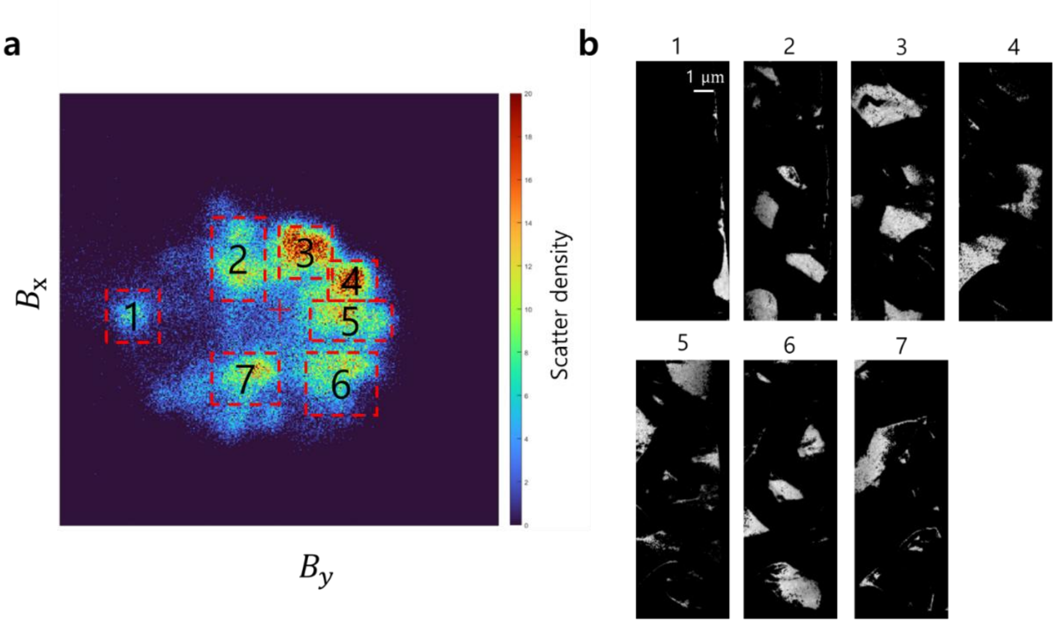

Figure S8: a 2D Histogram plot of vector components of the magnetic field (B_x vs. B_y) over the whole mapping area. The dash rectangles with numbers 1 to 7 are used for mapping. **b** Corresponding maps from the selections in **a** are shown.

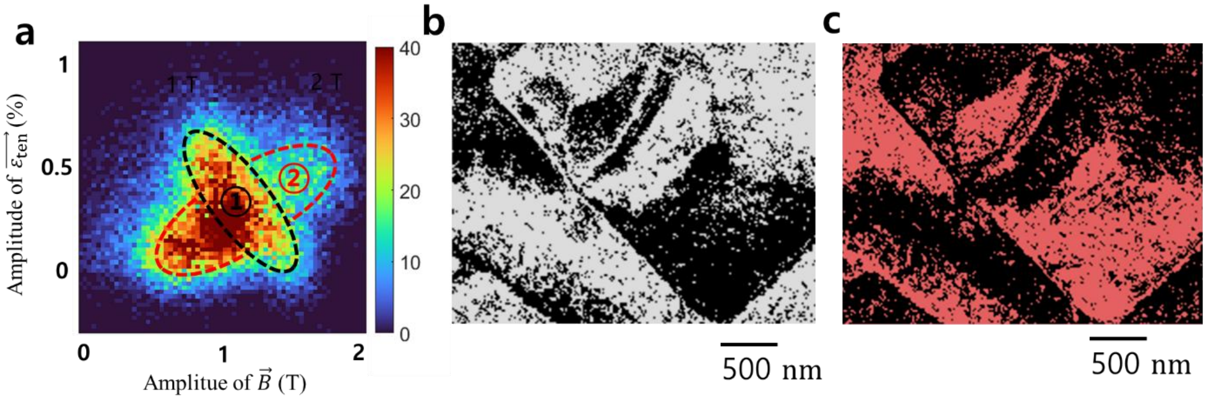

Figure S9: Correlative analysis of local strain and magnetization for a plastically deformed amorphous metallic alloy. **a** 2D distribution map of pixels according to $|\vec{\mathbf{B}}|$ and $|\vec{\boldsymbol{\varepsilon}}_{\mathbf{ten}}|$. It shows an interesting star-shaped feature with proportional (region ②) and inverse proportional (region ①) correlations. **b** and **c** Spatial distribution of map pixels contained in the components ① and ② in **a.**

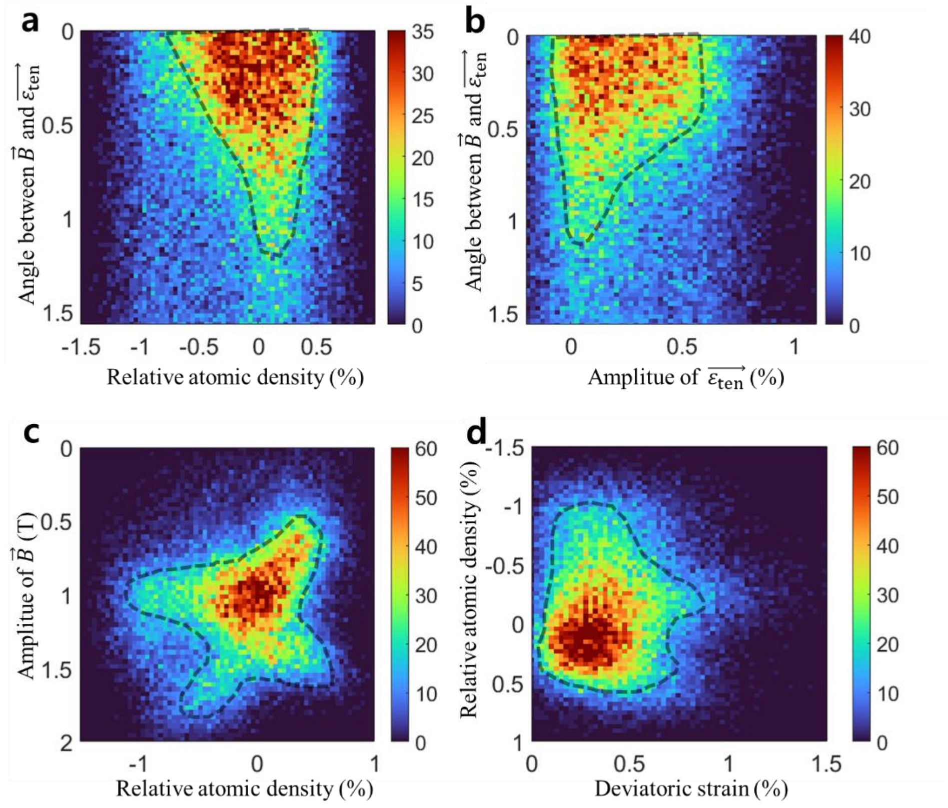

Figure S10: Exemplary density plots (2D histogram analysis) of **a** misorientation angle of $\vec{\boldsymbol{\varepsilon}}_{\mathbf{ten}}$ and $\vec{\mathbf{B}}$ and relative atomic density, **b** misorientation angle of $\vec{\boldsymbol{\varepsilon}}_{\mathbf{ten}}$ and $\vec{\mathbf{B}}$ and amplitude of $\vec{\boldsymbol{\varepsilon}}_{\mathbf{ten}}$, **c** amplitude of $\vec{\mathbf{B}}$ and relative atomic density, and **d** relative atomic density and deviatoric strain.

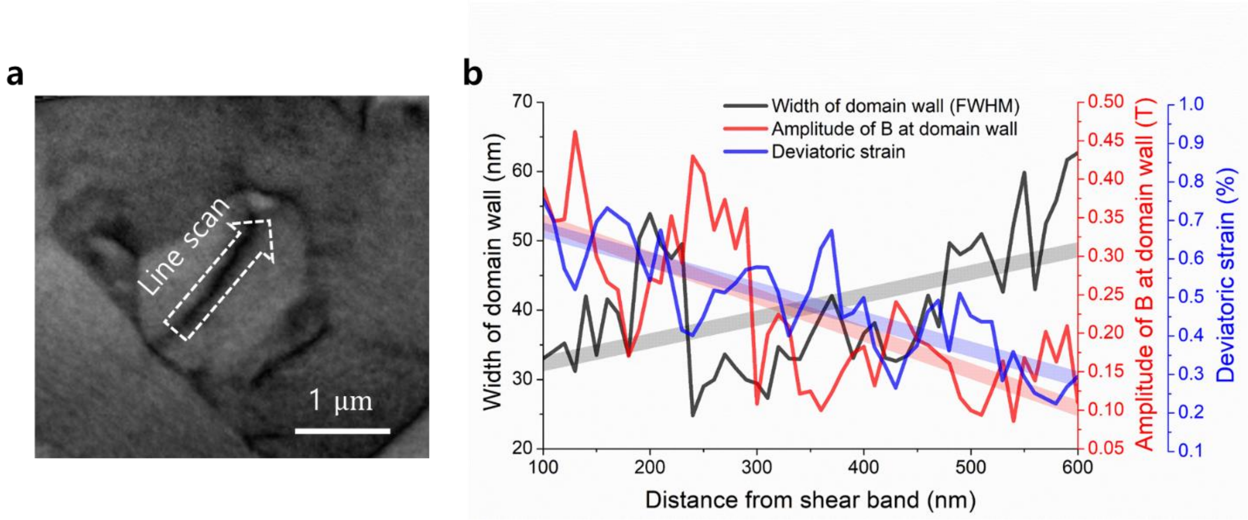

Figure S11: Correlative analysis across a domain wall. **a** An area of interest in $|\vec{\mathbf{B}}|$ map. **b** Line profiles of the quantities of $|\vec{\mathbf{B}}|$, $|\vec{\boldsymbol{\varepsilon}}|$ and deviatoric strain, taken across the domain wall.

References

1. Kang S, et al. Direct Observation of Quadrupolar Strain Fields forming a Shear Band in Metallic Glasses. *Advanced Materials* **35**, e2212086 (2023).
2. Mu X, et al. Unveiling the local atomic arrangements in the shear band regions of metallic glass. *Advanced Materials* **33**, 2007267 (2021).
3. Pan S, et al. Origin of splitting of the second peak in the pair-distribution function for metallic glasses. *Physical Review B—Condensed Matter and Materials Physics* **84**, 092201 (2011).
