# Supplementary material for: Large-angle Lorentz Four-dimensional scanning transmission electron microscopy for simultaneous local magnetization, strain and structure mapping
Source: Nat Commun. 2025 Feb 3;16:1305. doi: 10.1038/s41467-025-56521-6 (PMC11790882; doi:10.1038/s41467-025-56521-6)
Supplement: Supplementary file 2 — Description of Additional Supplementary Files [file 41467_2025_56521_MOESM2_ESM.pdf]

Description of Additional Supplementary Files

Supplementary Movie 1 - in-situ magnetization video of a plastically deformed amorphous metallic alloy by activating the objective lens

Supplementary Movie 2 - in-situ magnetization video of undeformed amorphous metallic alloy by activating the objective lens
